# Nitrogen limitation, toxin synthesis potential, and toxicity of cyanobacterial populations in Lake Okeechobee and the St. Lucie River Estuary, Florida, during the 2016 state of emergency event

**DOI:** 10.1371/journal.pone.0196278

**Published:** 2018-05-23

**Authors:** Benjamin J. Kramer, Timothy W. Davis, Kevin A. Meyer, Barry H. Rosen, Jennifer A. Goleski, Gregory J. Dick, Genesok Oh, Christopher J. Gobler

**Affiliations:** 1 School of Marine and Atmospheric Sciences, Stony Brook University, NY, United States of America; 2 Department of Biological Sciences, Bowling Green State University, Bowling Green, OH, United States of America; 3 Cooperative Institute for Great Lakes Research (CIGLR), University of Michigan, Ann Arbor, MI, United States of America; 4 Department of Earth and Environmental Sciences, University of Michigan, Ann Arbor, MI, United States of America; 5 USGS, Orlando, FL, United States of America; University of Wisconsin Milwaukee, UNITED STATES

## Abstract

Lake Okeechobee, FL, USA, has been subjected to intensifying cyanobacterial blooms that can spread to the adjacent St. Lucie River and Estuary via natural and anthropogenically-induced flooding events. In July 2016, a large, toxic cyanobacterial bloom occurred in Lake Okeechobee and throughout the St. Lucie River and Estuary, leading Florida to declare a state of emergency. This study reports on measurements and nutrient amendment experiments performed in this freshwater-estuarine ecosystem (salinity 0–25 PSU) during and after the bloom. In July, all sites along the bloom exhibited dissolved inorganic nitrogen-to-phosphorus ratios < 6, while *Microcystis* dominated (> 95%) phytoplankton inventories from the lake to the central part of the estuary. Chlorophyll *a* and microcystin concentrations peaked (100 and 34 μg L^-1^, respectively) within Lake Okeechobee and decreased eastwards. Metagenomic analyses indicated that genes associated with the production of microcystin (*mcyE*) and the algal neurotoxin saxitoxin (*sxtA*) originated from *Microcystis* and multiple diazotrophic genera, respectively. There were highly significant correlations between levels of total nitrogen, microcystin, and microcystin synthesis gene abundance across all surveyed sites (*p* < 0.001), suggesting high levels of nitrogen supported the production of microcystin during this event. Consistent with this, experiments performed with low salinity water from the St. Lucie River during the event indicated that algal biomass was nitrogen-limited. In the fall, densities of *Microcystis* and concentrations of microcystin were significantly lower, green algae co-dominated with cyanobacteria, and multiple algal groups displayed nitrogen-limitation. These results indicate that monitoring and regulatory strategies in Lake Okeechobee and the St. Lucie River and Estuary should consider managing loads of nitrogen to control future algal and microcystin-producing cyanobacterial blooms.

## Introduction

Climate change and eutrophication can promote the dominance of cyanobacteria among freshwater phytoplankton communities [1–3]. Human activities that enrich freshwater ecosystems with nutrients have been linked to the emergence of dense cyanobacterial blooms that can attenuate light and create hypoxic (low-oxygen) zones, deleteriously impacting benthic flora as well as pelagic and benthic fauna, respectively [4]. Shallow, non-stratifying lakes are especially vulnerable to excessive nutrient levels and the dominance of harmful cyanobacteria within phytoplankton communities [5, 6]. Many of the cyanobacterial genera that bloom under these conditions, including *Aphanizomenon*, *Dolichospermum (Anabaena*), *Cylindrospermopsis*, *Planktothrix*, and *Microcystis*, can be comprised of strains that produce toxins that can impact the health of aquatic life and humans [7–9]. Many *Microcystis* spp., for instance, can produce the hepatotoxin microcystin [10].

As harmful cyanobacterial blooms recur and/or intensify in freshwater bodies, it is important to identify the conditions that promote these events, as well as the causative cyanobacterial genera. It is traditionally assumed that phosphorus (P)-availability controls primary productivity in freshwater ecosystems [11, 12], and the proportion of cyanobacteria that comprise phytoplankton communities can be inversely correlated with total nitrogen-to-phosphorus (N:P) ratios [13]. The decrease in N:P can lead to the dominance of diazotrophic cyanobacterial genera capable of converting dinitrogen (N_2_) gas to ammonia (NH_3_) [14]. Recent meta-analyses, however, have demonstrated that the growth and toxin production of some non-diazotrophic cyanobacterial genera (e.g. *Microcystis* and *Planktothrix*) can be controlled by nitrogen (N) [15, 16]. In Lake Taihu, China, for instance, *Microcystis* spp. outcompete diazotrophs partly because of the supply of regenerated N and P from resuspended sediments in this shallow (~2 m mean depth) lake [5]. Other factors that may facilitate the dominance of non-N_2_ fixing cyanobacteria such as *Microcystis* over N_2_ fixers include reductions in water column irradiance [17] and elevated temperature [3, 18, 19].

Lake Okeechobee is the largest lake in the southeastern United States and has been subjected to eutrophication since the 1970s [20, 21]. The lake exhibits low submerged plant biomass, a benthic invertebrate community dominated by oligochaetes, and a phytoplankton community mainly comprised of cyanobacteria, all of which significantly limit the lake’s ability to sequester phosphorus [22]. While at least some regions of Lake Okeechobee were shown to exhibit N and P co-limitation in the early 1990s [23], Havens et al. (2016) reported that hurricanes in the mid-2000s led to some of the largest, most toxic blooms of *M*. *aeruginosa* ever observed in Lake Okeechobee, possibly due in part to the prolonged retention of soluble reactive phosphorus, inorganic nitrogen, and total suspended solids in the water column [24]. Ultimately, however, it is unclear which nutrient or combination of nutrients regulates these blooms, or whether nutrient limitation characteristics change over time and under different environmental conditions.

During the 2015–16 winter season, the Florida peninsula was subjected to unusually high temperatures, storm activity, and rainfall, corresponding to the concurrent El Niño event [25]. Consequently, the lake exhibited a dramatic increase in water level in early 2016 [26], necessitating the release of billions of gallons of water through canals, one of which led to the St. Lucie River and Estuary in the east. A dramatic, persistent increase in nitrate and decrease in salinity throughout the estuary made conditions optimal for a *Microcystis* bloom to develop [27]. This bloom lasted from May to mid-July and spread throughout this brackish ecosystem [27], leading Florida to declare a state of emergency due to socioeconomic impacts and human health concerns. In addition to the dominance of *Microcystis*, other potential toxin producers were identified [28]. For this study, transect surveys and experiments were performed from Lake Okeechobee through the St. Lucie River and Estuary and the Indian River Lagoon during and after this bloom event. The objective of the study was to characterize nutrient levels, the phytoplankton community, their toxins, and toxin synthesis potential via sequencing of genes associated with microcystin and saxitoxin synthesis. Furthermore, nutrient amendment experiments were conducted to assess limitations on algal and cyanobacterial growth. During and after the bloom event, it was determined that algal growth and toxicity were tightly coupled to N availability.

## Materials and methods

### Area of study and sample collection

**Fig 1:**
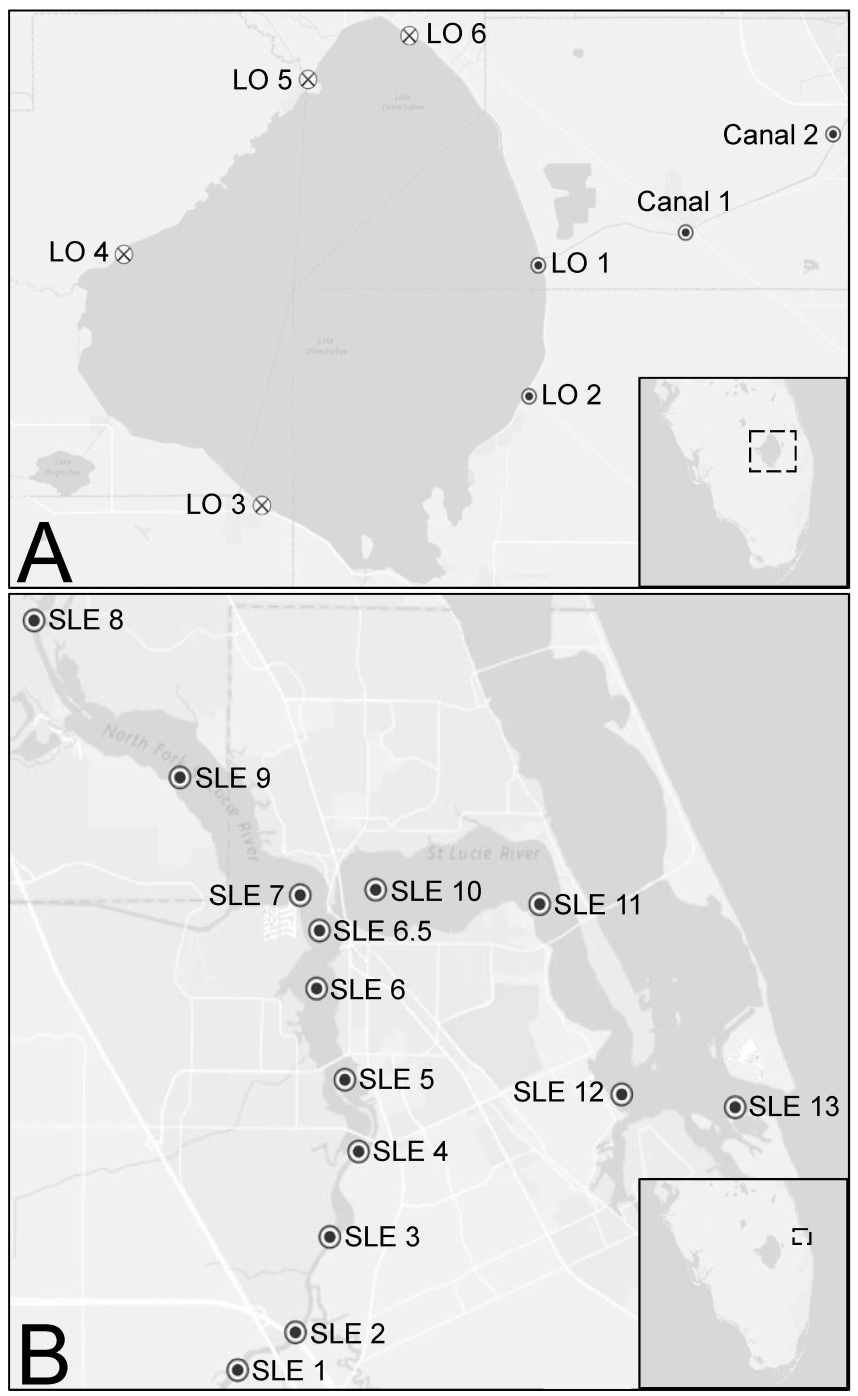
**Sampling sites around Lake Okeechobee (A) and on the St. Lucie River Estuary (B) in Florida, USA.** Sites sampled in both July and September 2016 (●) and September 2016 only (×) are represented. Inserts denote the general region sites were located on the peninsula.

Sampling and experiments were performed in July and September 2016, with samples collected from sites in Lake Okeechobee, the canal leading to the St. Lucie River (C-44), the St. Lucie River Estuary, and the Indian River Lagoon (Fig 1 and Table 1). Water samples from July (N = 20) and September (N = 22) were collected over the course of 3 and 4 days, respectively. Waterfront areas were accessed from public access points and the collection of small volumes of water is not a regulated activity in Florida. Temperature (°C), salinity (PSU) and dissolved oxygen (O_2_) concentrations (mg L^-1^) were measured at 22 sites using a YSI 556 probe (Table 1). Surface water was collected from seven sites that represented a salinity gradient across the system to characterize the phytoplankton community, their toxin synthesis potential, as well as toxin and nutrient concentrations. At four sites in July and three sites in September, large volumes of water from Lake Okeechobee to the river estuary were collected and nutrient amendment experiments were performed. For each of the seven full sampling sites, samples were collected and analyzed according to previously established protocols described below. For enumeration of phytoplankton, duplicate samples were preserved in Lugol’s iodine (5% final concentration) and microscopically quantified to at least the genus level based on morphological traits such as cell dimension, arrangement of cells in colonies or filaments, and the presence of specialized structures (i.e., aerotopes, heterocytes, akinetes). For the precise volume of the subsample for counting, a 4-place balance (Ohaus Explorer EX224, Ohaus Corporation, Parsippany, New Jersey, USA) was used (typically 0.0175 mL or less) and dispersed under a 22 mm^2^ cover slip. This method allows samples to be examined at 400x with an Olympus BX51 research microscope (Olympus America, Waltham, Massachusetts, USA); random strip-counts of known width allowed a precise calculation of the volume counted to obtain an accurate cell count per unit volume of the original sample.

**Table 1:** GPS coordinates as well as the collection dates (Month / Day) as well as the dissolved oxygen (mg L^-1^), temperature (°C), and salinity (PSU) levels from the July and September 2016 transects.

SITES			JULY 2016				SEPTEMBER 2016			
ID	General Location	Coordinates (LAT/LONG)	Month / Day	O_2_ (mg L^-1^)	°C	PSU	Month / Day	O_2_ (mg L^-1^)	°C	PSU
LO 1	Eastern Shore	26.864296, -80.63255	7/9*	7.5	32.4	0.1	9/26*	5.6	30.9	0.2
LO 2	Eastern Shore	26.984979, -80.620918	7/10	6.1	32.4	0.2	9/26*	6.4	30.1	0.2
LO 3	Southwestern Shore	26.760595, -80.918512	-	-	-	-	9/28	3.6	29.6	0.2
LO 4	Western Shore	26.992225, -81.067107	-	-	-	-	9/28	1.7	30.1	0.2
LO 5	Northern Shore	27.147202, -80.869267	-	-	-	-	9/28	3.4	30.2	0.1
LO 6	Northern Shore	27.191742, -80.76324	-	-	-	-	9/29	2.7	26.9	0.1
Canal 1	C-44 Canal	27.012359, -80.455056	7/10	6.5	33.9	0.2	9/29	5.8	29.7	0.2
Canal 2	C-44 Canal	27.09528, -80.296074	7/10	3.6	32.8	0.2	9/29	4.8	29.4	0.2
SLE 1	SF	27.11349, -80.28313	7/8, 7/9*	6.1	32.6	0.2	9/26*	6.4	30.0	0.2
SLE 2	SF	27.12091, -80.26969	7/8	6.6	32.2	0.2	9/27	6.1	29.8	0.2
SLE 3	SF	27.13966, -80.261706	7/8	7.7	33.3	0.2	9/27	5.8	29.9	0.2
SLE 4	SF	27.15646, -80.25502	7/8	8.5	34.1	0.2	9/27	5.7	29.9	0.2
SLE 5	SF	27.17057, -80.25821	7/8	6.7	33.1	0.2	9/27	5.4	29.8	0.2
SLE 6	ME	27.18850, -80.26478	7/8	8.7	32.9	2.2	9/27	5.5	29.8	0.2
SLE 6.5	ME	27.19989, -80.264114	7/9*	6.9	33.5	3.2	9/26*	5.3	30.2	0.8
SLE 7	ME	27.20684, -80.26859	7/8	5.9	33.1	3.6	9/27	7.4	31.0	1.8
SLE 8	NF	27.26080, -80.33047	7/8	6.2	33.9	0.5	9/27	4.8	29.1	0.4
SLE 9	NF	27.22998, -80.29655	7/8	8.2	34.1	0.7	9/27	5.6	31.3	0.8
SLE 10	ME	27.20792, -80.25105	7/8, 7/9*	7.8	33.7	7.4	9/26	5.2	30.4	4.2
SLE 11	IRL	27.20509, -80.21291	7/8	6.8	32.6	12.6	9/27	5.5	30.6	5.7
SLE 12	IRL	27.16769, -80.19385	7/8	6.8	31.1	27.5	9/27	5.6	30.0	10.3
SLE 13	SLI	27.16516, -80.16748	7/8	7.0	30.1	32.0	9/27	5.6	29.7	6.0

LO = Lake Okeechobee, Canal = drainage canal connecting the lake to the river estuary, SLE = Saint Lucie Estuary. Sites LO 3–6 were not included until the September 2016 transect.

* = dates when water was collected for nutrient amendment experimentation. General locations of sampling sites are also given, emphasizing from which lake shoreline (LO), canal (C-44), and estuary branch (SF = South Fork, ME = Middle Estuary, NF = North Fork, IRL = Indian River Lagoon, SLI = St. Lucie Inlet) samples were collected

For toxin analyses, 10 mL of whole water was stored at -20°**C** prior to quantification. Microcystin and saxitoxin concentrations were quantified using Abraxis enzyme-linked immunosorbent assays (ELISA) following the manufacturer’s protocols. Prior to running a microcystin ELISA, thawed samples were lysed and filtered using materials provided by Abraxis. ELISA limits of detection for microcystin and saxitoxin were 0.15 and 0.02 μg L^-1^, respectively. As the microcystin antibody provided in the Abraxis ELISA kit specifically binds to congeners with the β amino acid (ADDA) group, toxin concentrations were reported as microcystin–ELISA equivalents. Duplicate 20 mL, whole water samples were collected and stored frozen for total nutrient analyses whereas duplicate dissolved nutrient samples were passed through pre-combusted (450°C for 2 h) glass fiber (GFF) filters and stored frozen. GFF filters were also stored frozen until chlorophyll *a* was extracted with 5 mL 90% acetone and quantified to approximate algal biomass on a Turner Designs fluorometer. Concentrations of total N, total P, nitrite and nitrate (NO_2_^-^+NO_3_^-^), ammonia (NH_3_), and soluble reactive phosphorus (SRP)^-^ were quantified on a Lachat Instruments autoanalyzer [29]. During the September transect, a Fluoroprobe (bbe Moladaenke) was used to quantify the relative abundance of green algae, cyanobacteria, and diatoms, which were distinguished based on the spectral characteristics of their photosynthetic accessory pigments [30–32], in triplicate.

During the July transect, 20 mL of water was passed through a 2 μm polycarbonate filter (Millipore) and stored at -80°**C**. Frozen sample filters were thawed at room temperature before being cut into small strips in a sterile petri plate using a flame sterilized blade. Filter strips were then placed in a 2 mL microcentrifuge tube for DNA extraction using a Qiagen DNeasy® Blood and Tissue Kit (Qiagen, Hilden, Germany). Briefly, samples were incubated with 100 μL Qiagen ATL tissue lysis buffer, 300 μL Qiagen AL lysis buffer, and 30 μL proteinase K at 56°C for 1 h with agitation, followed by vortexing at maximum speed for 10 minutes. Lysates were homogenized with a QiaShredder™ spin-column before purification according to the DNeasy® protocol. DNA quantity and quality were assessed using a NanoDrop Lite (Thermo Fisher Scientific, NanoDrop Products, Wilmington, DE, USA).

### Analysis of toxin gene presence and abundance

#### Multiplex qPCR

Genes indicative of the genetic potential to produce microcystin/nodularin (*mcyE*), saxitoxin (*sxtA*) and cylindrospermopsin (*cyrA*) were enumerated using a commercially available multiplex qPCR kit (Phytoxigene CyanoDTec™ Toxin Genes Test; Diagnostic Technology, Sydney, Australia) modeled after the multiplex qPCR assay described in Al-Tebrineh et al. (2012). Briefly, molecular grade H_2_O (80 μL) was added to each tube of a CyanoDTec™ cyanotoxin detection kit and processed following kit directions. A synthetic standard of known toxin gene copy (Diagnostic Technology) was assayed in serial dilutions to generate a standard curve spanning five orders of magnitude (100–1,000,000 copies) for each target toxin gene. Amplification, per the kit protocol, was conducted in 96-well plates on a 7500 Fast Real-Time PCR system (Applied Biosystems, Waltham, MA, USA) in a total volume of 25 μL. Each sample was run in duplicate. Gene copies in each reaction were calculated using the Applied Biosystems software and back-calculated to copies mL^-1^ [33].

#### Metagenomic analysis

Samples were submitted to the University of Michigan DNA Sequencing Core for sequencing on the Illumina® HiSeq™ platform (4000 PE 150, Illumina, Inc., San Diego, CA, USA). Sequence reads were checked for quality using FASTQC version 0.10.0 (http://www.bioinformatics.babraham.ac.uk/projects/fastqc/)) and de-replicated (100% identity over 100% of length) before adapter removal using Scythe and read trimming using Sickle. Sequences were then assembled *de novo* with the iterative de Bruijn graph approach for uneven sequencing depths (IDBA-UD) with the following parameters: minimum kmer size 52, maximum kmer size 92, step size 8, minimum contig 500.

Sequence reads remaining after read trimming as well as assembled scaffolds were searched for microcystin and saxitoxin production genes using BLASTN against a database complete target sequence for the *mcyE* and *sxtA* standards in the aforementioned multiplex qPCR kit, provided by Diagnostic Technology (Belrose, Australia). Reads and scaffolds with BLAST hits with > 80% query coverage and > 97% ID were then put through a standard nucleotide BLAST against the NCBI nucleotide collection (nr/nt) for cyanobacteria for taxonomic identification. The coverage of toxin genes was calculated by mapping trimmed reads against assembled scaffolds using BWA mapper with default parameters and then calculating coverage with bedtools’ multicov function.

In addition to the aforementioned multiplex qPCR target sequences, the full primer and probe sequences used in each of the *mcyE* and *sxtA* qPCR analyses were searched for in all samples’ assembled scaffolds using BLASTN. To be considered positive for saxitoxin production the forward primer, sequence probe, and reverse primer all needed to hit the same scaffold with 100% identity for the entire lengths of the primer/probe and be aligned in order. All scaffolds from samples positive for putative saxitoxin production were then binned into putative taxonomic groups with emergent self-organizing maps (ESOM) of tetranucleotide frequencies (Robust ZT transformation) using Databionics ESOM Tools (http://databionic-esom.sourceforge.net). Reference genomes were included for ESOM training and binning reference including two species of *Dolichospermum* (*Anabaena*), and one species each of *Aphanizomenon*, *Cyanobium*, *Oscillatoria*, *Planktothrix*, *Pseudanabaena*, and *Synechococccus*. Only contigs longer than 3000 kb were considered for ESOM binning and contigs longer than 5 kb were cut to fit into the 3–5 kb window. Other training parameters for the ESOM were: K-batch algorithm (k = 0.15%) for 40 training epochs, standard best match search method with a local best match search radius of 8, a Gaussian weight initialization, Euclidean data space function, a starting training radius of 213 with linear cooling to 1, and a starting learning rate of 0.5 with linear cooling to 0.1. Bins containing scaffolds with BLAST hits to *sxtA* genes were identified and all scaffolds in that bin were extracted for taxonomic identification using a combination of BLASTN of contigs against the Silva SSU Database version 119 and a standard nucleotide BLAST against the NCBI nucleotide collection (nr/nt).

### Nutrient amendment experimental design

During sample collection for the July and September transects, surface water was collected from Lake Okeechobee (station LO 1), the canal connecting Lake Okeechobee and the St. Lucie Estuary (SLE 1) and two stations in the main stem of the St. Lucie Estuary (SLE 6.5, and SLE 10). Sample water was transferred into triplicate Nalgene, polycarbonate bottles (250 mL in July, 1 L in September) and amended with either 20 μM ammonium (NH_4_^+^), 2 μM PO_4_^3-^, or NH_4_^+^ and PO_4_^3-^ (20 and 2 μM, respectively). Bottles were incubated in the eastern extent of the St. Lucie River. As *Microcystis* descends deeper into the water column in response to elevated irradiance [34], likely to avoid the harmful effects of elevated UV levels [35], bottles were placed under one layer of neutral density screening, mimicking light levels~ one meter below the surface. After 24 h, samples were collected for the analysis of chlorophyll *a* and phytoplankton quantification in July, whereas during September, samples for chlorophyll *a* analysis were collected after 72 h and were also analyzed via the Fluoroprobe after 48 h. Chlorophyll *a* values from both experiments were used to approximate growth rate (day^-1^) at specific time points during experimentation using the formula:

$$\frac{ln*(\frac{N_{t}}{N_{0}})}{t}$$ (1)

where N_t_ is the final biomass, N_o_ is the initial biomass, and t is time in days.

### Nitrogen fixation and statistics

During September transect sampling, N_2_-fixation rates were measured using an acetylene reduction assay [36]. To quantify rates, 5 mL of sample water was placed in gas-tight 10 mL glass septum vials, injected with 500 μL of acetylene (C_2_H_2_) via a gas-tight, glass syringe, and incubated in the field. After 4 h, 90 mM zinc acetate (C_4_H_6_O_4_Z_n_) was added to each vial to preserve samples and cease all biological processes [37]. Samples were stored at room temperature prior to ethylene (C_2_H_4_) quantification using a Trace 1300 Gas Chromatograph (Thermo Fisher Scientific). The amount of ethylene produced was quantified using standards and the amount of N_2_ fixed was determined assuming a 4:1 ratio [38].

A one-way analysis of variance (ANOVA) was performed using SigmaPlot (Version 11.0) to compare differences among transect sites whereas experimental treatments at specific time points for all parameters were compared via a two-way ANOVA where nitrogen and phosphorus were the main effects (*p* < 0.05).

## Results

### Lake-to-ocean transect, July

**Fig 2:**
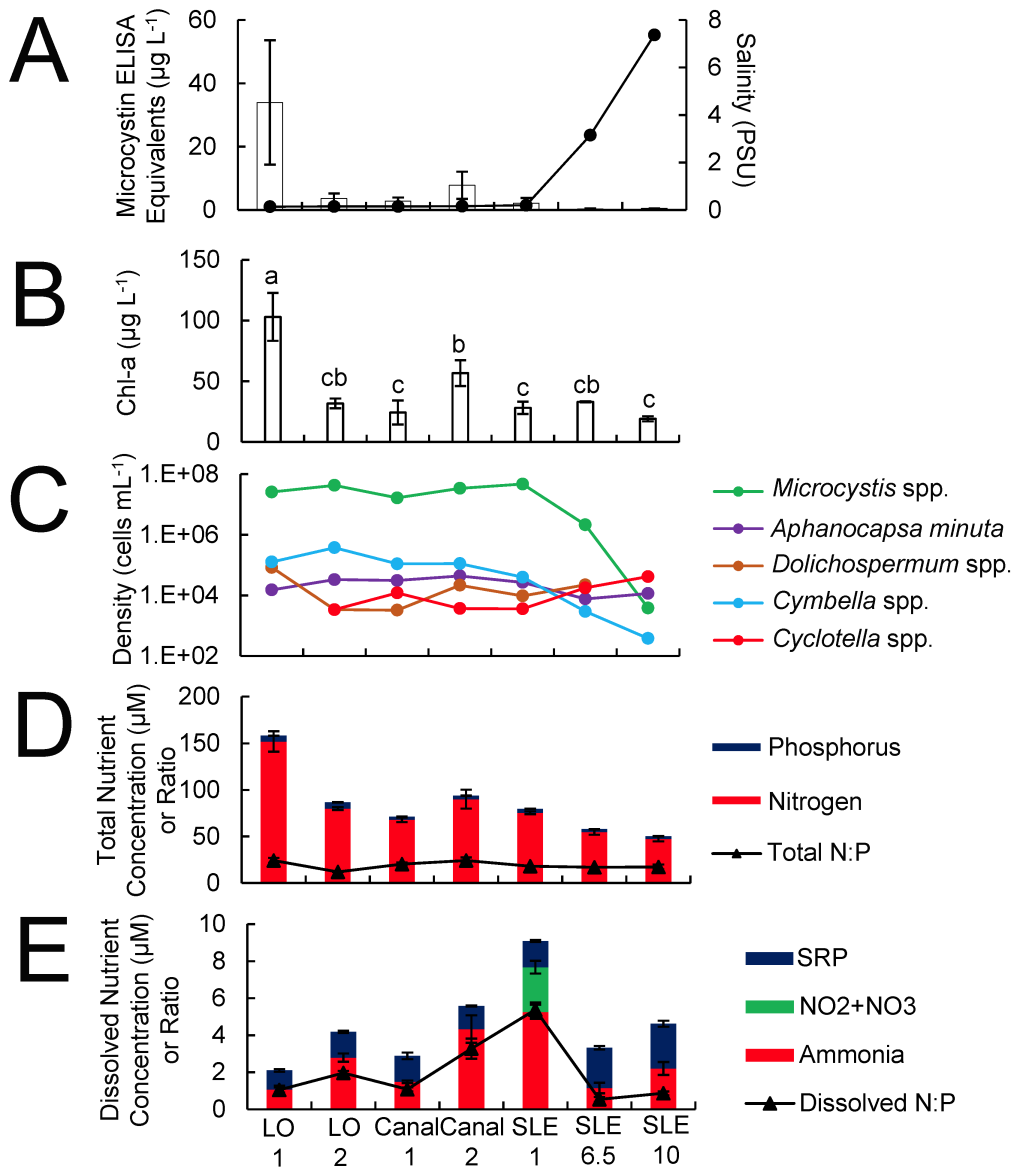
July 2016 transect data of sites that represented a strong salinity gradient. Included are microcystin and salinity values (A), chlorophyll *a* concentrations (B), densities of the five most abundant phytoplankton (C), and total (D) and dissolved (E) inorganic nitrogen and phosphorus concentrations and ratios. Algal densities are represented on the log scale in C. Error bars denote standard deviations.

**Fig 3:**
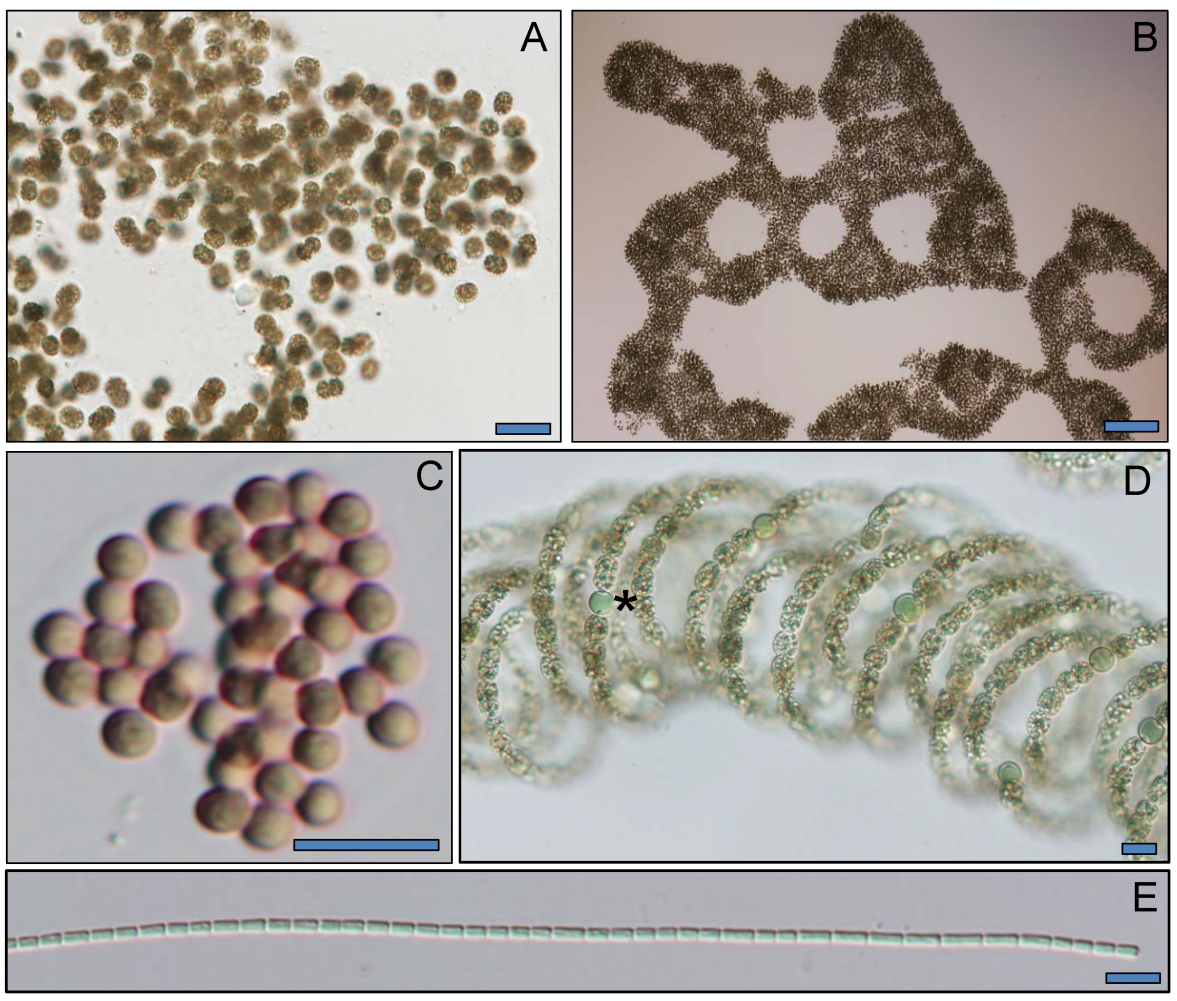
Cyanobacterial taxa abundant in the 2016 summer transect. *Microcystis aeruginosa*, bar is 20 μm (A) and 100 μm (B). *Aphanocapsa grevillei*, bar is 10 μm (C). *Dolichospermum circinale*, bar is 10 μm and asterisk (*) denotes heterocyst (D). *Pseudanabaena* spp., bar is 10 μm (E).

Across the transect from Lake Okeechobee and the St. Lucie River, salinity levels were ≤ 0.2 PSU except for two eastern sites within the St. Lucie River where levels were 3–8 PSU (Fig 2A). Chlorophyll *a* levels were elevated (> 20 μg L^-1^), with levels exceeding 100 μg L^-1^ at LO 1 (Fig 2B). Cyanobacteria numerically dominated (> 95%) the phytoplankton community, with *Microcystis* making up the majority (> 95%) of cells at all sites (106–10^7^ cells mL^-1^), save for site SLE 10 (Figs 2C and 3). Other cyanobacteria present at lower densities (10^3^–10^5^ cells mL^-1^) at most sites included *Dolichospermum* (*Anabaena*) and *Aphanocapsa* spp. (Figs 2C and 3). The diatoms *Cyclotella* spp. and *Cymbella* spp. were present throughout the transect at densities comparable to the two non-*Microcystis* cyanobacteria (10^2^–10^5^ cells mL^-1^; Fig 2C).

Across the transect, total nitrogen concentrations were higher (40–160 μM) than total phosphorus values (2–7 μM), and the total N:P ratio ranged from 12–25 (Fig 2D). Total nitrogen and phosphorus levels generally declined from Lake Okeechobee through the St. Lucie River Estuary (Fig 2D). Dissolved nitrogen (NH_3_) levels (1–6 μM) were much lower than total nitrogen levels, though dissolved phosphorus (SRP) levels (1–3 μM) were comparable to total phosphorus levels. Furthermore, nitrate levels were largely undetectable, save at site SLE 1 (Fig 2E). The dissolved nitrogen-to-phosphorus ratio (DIN:DIP) was well below Redfield, ranging from 0.5–5.5 and averaging ~ 2 (Fig 2E).

Microcystin ELISA equivalent levels were highest in Lake Okeechobee (3.6–34 μg L^-1^) but lower in the St. Lucie River Estuary (0.3–7.8 μg L^-1^) (Fig 2A). Interestingly, regions along the transect that exhibited the highest *Microcystis* spp. concentrations (LO 2 and SLE 1), did not correspond to the highest microcystin concentrations (Fig 2A and 2C). Saxitoxin concentrations were at or below the methodological detection limit in all samples.

#### Multiplex qPCR

**Fig 4:**
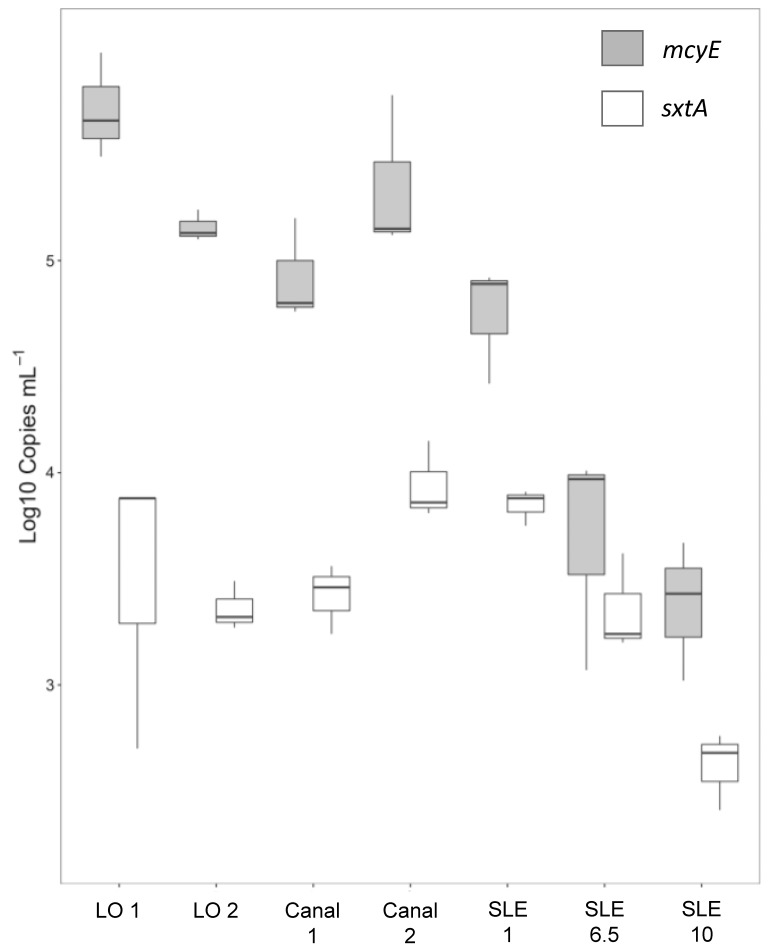
*mcyE* (white) and *sxtA* (shaded) gene abundances (copies mL^-1^) at sites along the July 2016 transect. Values are log-transformed and represented in boxplots.

At all stations sampled during July 2016, both *mcyE* and *sxtA* were above the limit of quantification (Fig 4). *mcyE* gene copies ranged from 2.8 ± 1.8 x 10^3^ mL^-1^ to 3.8 ± 1.0 x 10^5^ mL^-1^, whereas *sxtA* copies ranged from 0.44 ± 0.2 x 10^3^ mL^-1^ to 7.6 ± 0.04 x 10^3^ mL^-1^ (Fig 4). For both *mcyE* and *sxtA*, the highest gene copy levels were detected at site LO 1 and decreased throughout the St. Lucie River sites as salinity increased (Figs 2A and 4).

#### Putative producers of microcystins and saxitoxins

There were positive BLASTN hits for the *mcyE* primer, a subunit of the microcystin synthesis gene operon, in the reads and assembled scaffolds from all samples collected (Fig 4). Scaffold BLASTN hits were limited to a %ID greater than 98%, which corresponded with a *mcyE* primer coverage of 55–82%, and run against the NCBI cyanobacteria database using BLASTN. All scaffolds with a positive *mcyE* hit matched to *Microcystis*, and for all but one sample (SLE 10) the raw coverage of *mcyE* was > 100X. As *Microcystis aeruginosa* was the dominant, potentially toxic cyanobacterium in the Lake Okeechobee-St. Lucie waterway at the time, it was the likely microcystin producer.

Positive BLASTN hits for the *sxtA* primer, a subunit of the saxitoxin synthesis operon, were found in the reads of all samples with greater than 98% ID but covering 80% or less of the *sxtA* primer. When the same *sxtA* primer was aligned to assembled scaffolds there were only three hits: one each to sample SLE 1, SLE 6.5, and Canal 1 with 100% ID and 80% coverage. The *sxtA* genes identified in these three samples had raw read coverages of 18, 27, and 9X, respectively. Hits to only these three samples were verified by using the full forward primer, probe, and reverse primer compliment which again identified the same three samples and scaffolds. Both primers and the probe were found to align in order along the previously identified scaffolds. The scaffolds with these hits were compared to the NCBI cyanobacteria database, which returned putative taxonomies of *Dolichospermum* (*Anabaena*), *Aphanizomenon*, *Lyngbya*, or *Cylindrospermopsis*.

To increase taxonomic identification, similar scaffolds were binned and extracted by ESOM and compared to both SSU and nucleotide databases using BLASTN. Within the identified bin for each sample, putative taxonomy was assigned using the BLASTN bit score (minimum 1000). For all three samples, the highest bit scores were consistently associated with *Dolichopsermum*, indicating that this genus was likely the dominant saxitoxin producer in the Lake Okeechobee-St. Lucie waterway.

### Nutrient effects on phytoplankton populations, July

**Fig 5:**
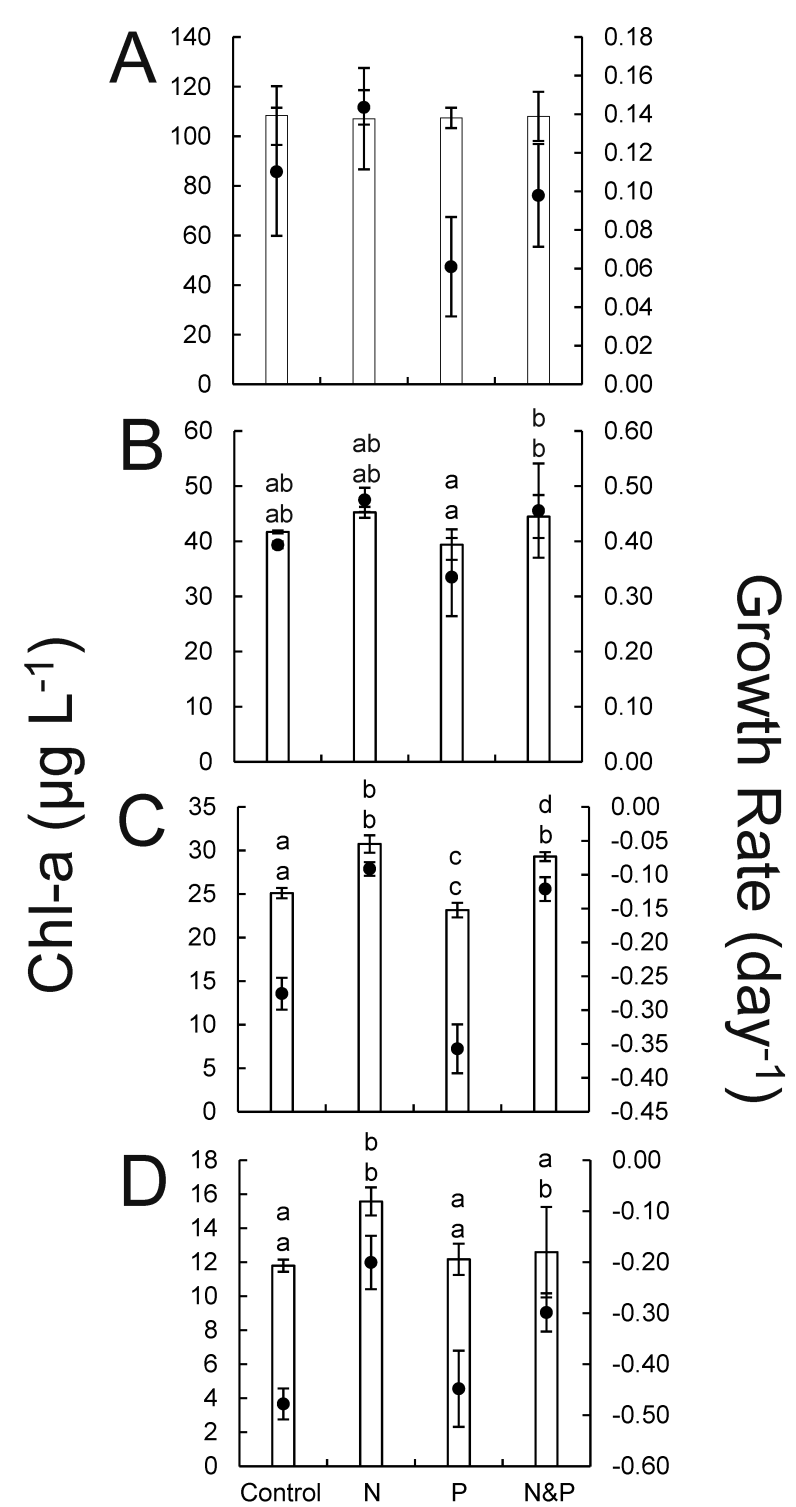
**Chlorophyll *a* (bar) and specific growth rate (black circle) data from July 2016 24 h nutrient amendment experiments from samples collected from sites LO 1 (A), SLE 1 (B), SLE 6.5 (C), and SLE 10 (D). Error bars denote standard deviations.** Statistical significance (*p* < 0.05) among sites is denoted by different letter combinations. Top and bottom letters above bar graphs correspond to statistical differences in chlorophyll *a* and specific growth rate, respectively.

In all estuarine experiments, the addition of NH_4_^+^ yielded the highest levels of phytoplankton biomass after 24 h (Fig 5). For the experiment using lake water, differences among treatments were not significant (Fig 5A). However, for the experiments performed at SLE 6.5 and SLE 10, the addition of NH_4_^+^ with or without PO_4_^3-^ yielded biomass levels and/or growth rates that were significantly greater than the control treatment (*p* < 0.05; Fig 5C and 5D).

### Lake-to-ocean transect, September

**Fig 6:**
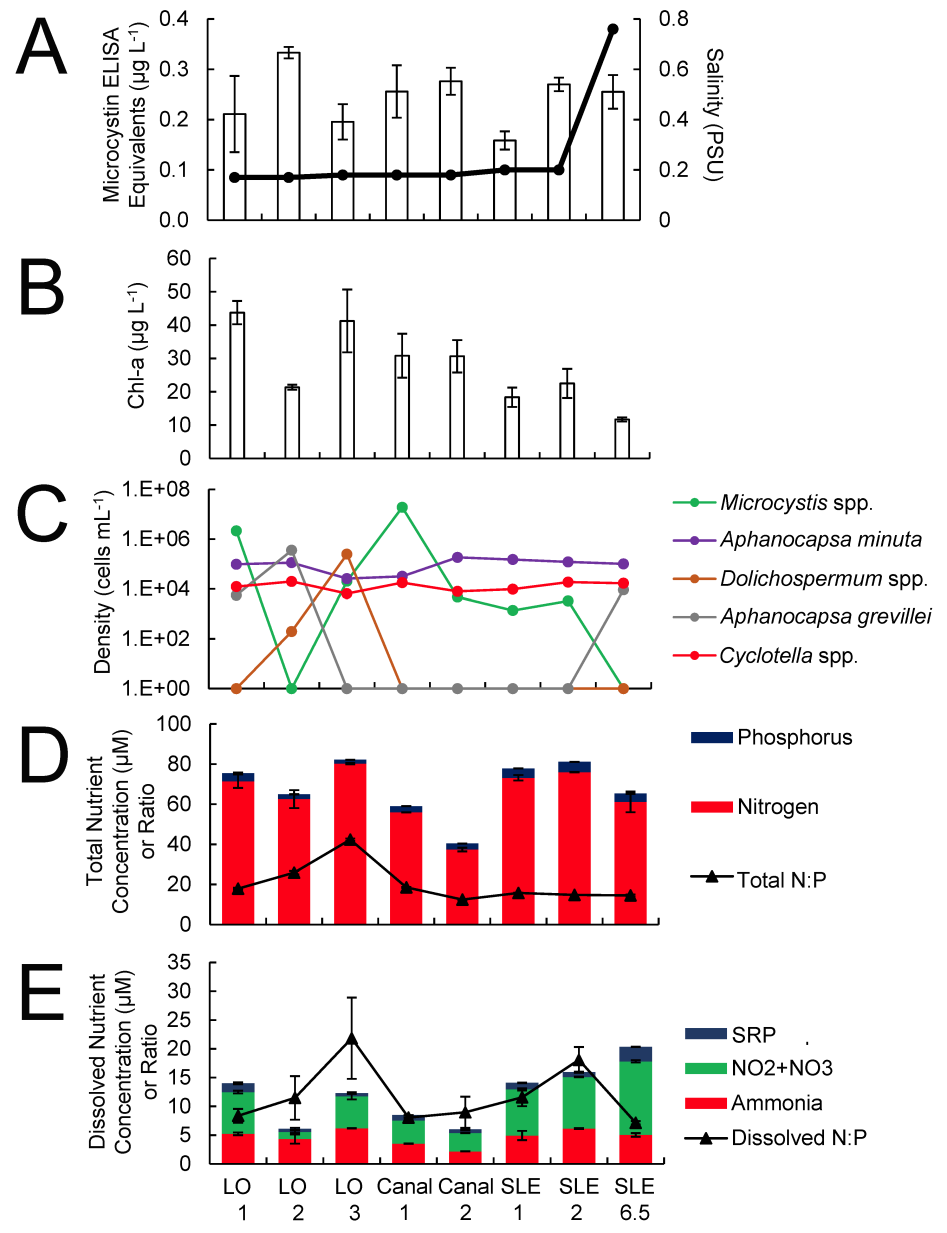
September 2016 transect data of sites that represented a strong salinity gradient. Included are microcystin and salinity values (A), chlorophyll *a* concentrations (B), densities of the five most abundant phytoplankton (C), and total (D) and dissolved (E) inorganic nitrogen and phosphorus concentrations and ratios. Algal densities are represented on the log scale in C. Error bars denote standard deviations.

**Fig 7:**
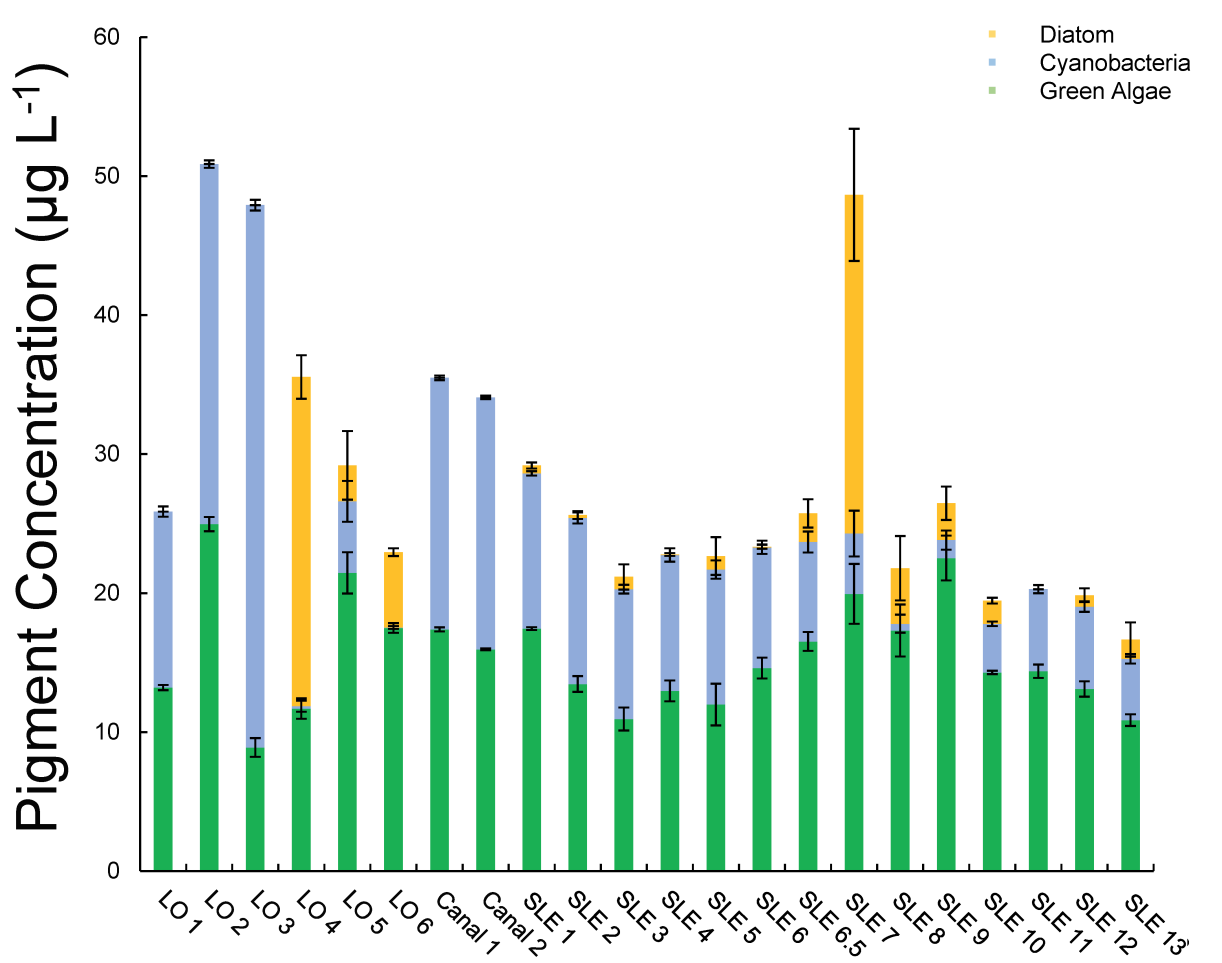
September 2016 diatom, cyanobacteria, and green algal pigment concentrations from all transect sites. Error bars represent standard deviations.

Salinity levels were near zero throughout most of the September transect, with the site furthest east reaching ~ 1 PSU (Fig 6A). Chlorophyll *a* levels across the transect were within the range found in July, generally between 11–44 μg L^-1^ (Fig 6B). Differential algal pigment analyses revealed that Lake Okeechobee had higher (> 20 μg L^-1^) cyanobacterial concentrations than estuarine sites (Fig 7), suggesting that salinity (Table 1) partially regulated the biomass of this phytoplankton group. Compared to cyanobacteria, diatom pigment concentrations were low (< 10 μg L^-1^) throughout the Lake Okeechobee–St. Lucie River waterway except at sites LO 4 and SLE 7 (> 20 μg L^-1^; Fig 7). Green algae exhibited a lower variation (9–25 μg L^-1^) in abundance and were the dominant group within the estuarine region (Fig 7).

Lake site LO 1 and the canal site closest to Lake Okeechobee, Canal 1, had phytoplankton cell densities (10^6^–10^7^ cells mL^-1^) and diversity (*Microcystis* spp. ≥ 95% of cell densities) most similar to sites surveyed during the July transect. Generally, however, fall transect cell densities were lower (≤ 10^5^ cells mL^-1^) and cyanobacteria were dominated by *Dolichospermum* (*Anabaena*) spp. and *Aphanocapsa* spp. (Fig 6C). While *Cymbella* spp. abundance was considerably lower relative to July, *Cyclotella* spp. abundances were similar (Fig 6C). Total nitrogen concentrations were higher (37–81 μM) than total phosphorus concentrations (1–6 μM), and the total N:P ratio ranged from 12–43 (Fig 6D). Dissolved nutrient concentrations were generally higher in September (5–20 μM) than in July (2–9 μM), while DIN:DIP was still below Redfield, ranging from 7–12 except at sites LO 3 and SLE 2 (> 16) (Fig 6E). At these two sites, *Microcystis* spp. concentrations were < 10^5^ cells mL^-1^ and not the dominant cyanobacteria in the algal community (Fig 6C). Furthermore, NO_3_^-^ concentrations were higher and measurable at all sites in September. Microcystin concentrations were uniformly low in September, ranging from 0.15 to 0.35 μg L^-1^, and did not exhibit a spatial trend (Fig 6A). N_2_-fixation was below detection throughout the transect.

### Nutrient effects on phytoplankton, September

**Fig 8:**
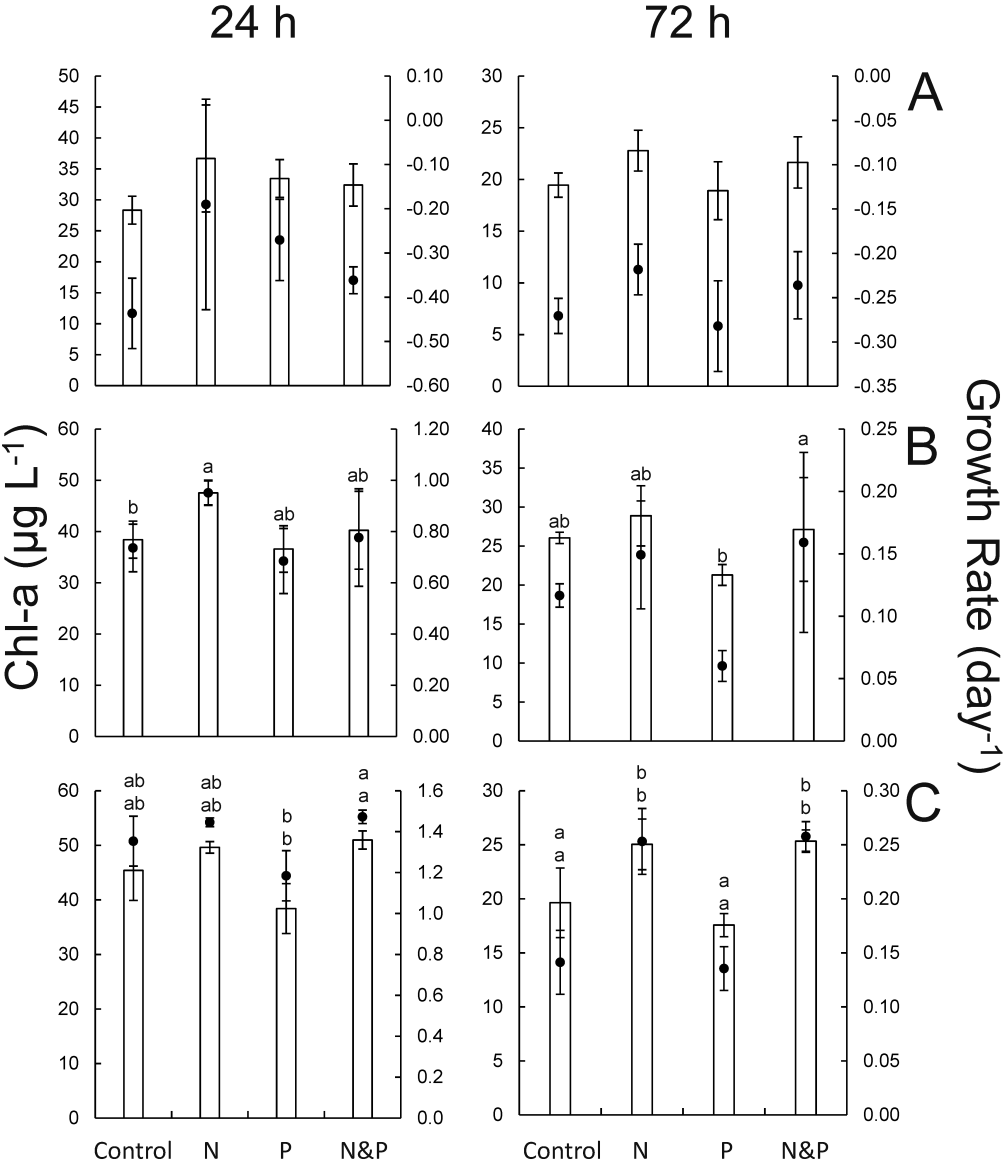
Chlorophyll *a* (bar) and specific growth rate (black circle) data at 24 and 72 h from the September 2016 nutrient amendment experiment. Samples were collected from sites LO 1 (A), SLE 1 (B), and SLE 6.5 (C). Error bars denote standard deviations. Statistical significance (*p* < 0.05) among sites is denoted by different letter combinations. Top and bottom letters above bar graphs correspond to statistical differences in chlorophyll *a* and specific growth rate. For B, statistical differences in chlorophyll *a* and growth rate were only present at 24 and 72 h, respectively.

**Fig 9:**
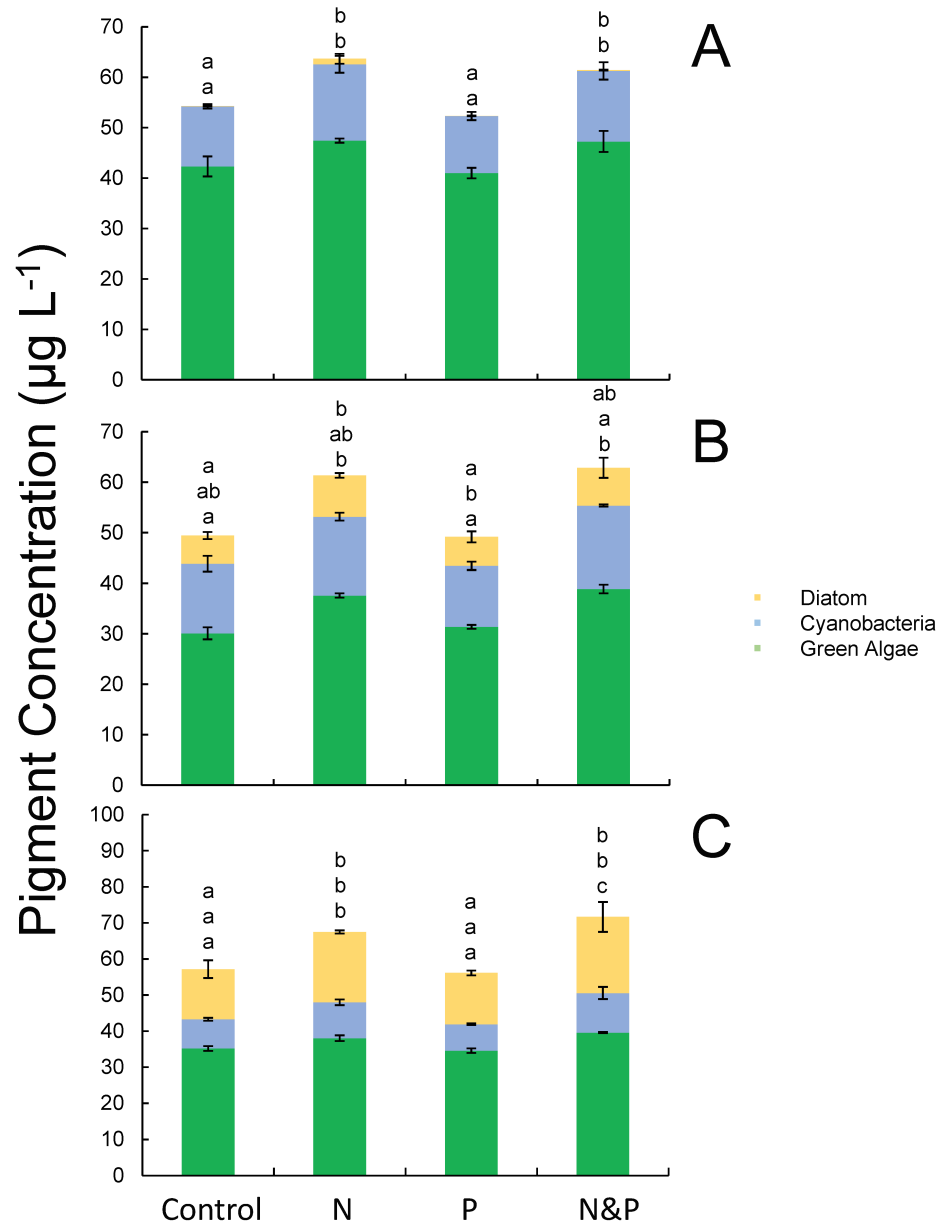
Diatom, cyanobacteria, and green algal pigment concentrations at 48 h from the September 2016 nutrient amendment experiment. Samples were collected from sites LO 1 (A), SLE 1 (B), and SLE 6.5 (C). Error bars represent standard deviations. Statistical significance (*p* < 0.05) of specific pigments among sites is denoted by different letter combinations. Letters are ordered relative to the order of parameters listed in the legend. For A, only cyanobacterial and green algal pigment concentrations exhibited statistical differences among treatments.

During the September experiments, nitrogen additions yielded the highest chlorophyll *a* concentrations in every experiment (Fig 8). At lake site LO 1, ammonium alone yielded significantly higher cyanobacterial and green algal pigment concentrations relative to untreated control bottles (*p* < 0.05; Fig 9A). At the estuary site SLE 1, chlorophyll *a*, green algae, and diatom pigment concentrations significantly increased in water treated with NH_4_^+^-only (*p* < 0.05; Figs 8B and 9B). For the experiment using water from site SLE 6.5, the addition of ammonium with or without phosphate yielded the highest levels of algal biomass and growth rate with significantly higher growth rates relative to the control group after 72 h (*p* < 0.05; Fig 8C). Cyanobacteria, green algae, and diatoms all exhibited similar responses to nutrient amendments at SLE 6.5, with samples treated with NH_4_^+^ exhibiting significantly higher pigment concentrations relative to control and PO_4_^3—^only treatments (*p* < 0.05; Fig 9C).

## Discussion

While toxic cyanobacterial blooms have become commonplace in freshwater bodies around the globe, the Lake Okeechobee and the St. Lucie River and Estuary state of emergency bloom event in 2016 was distinctive in several ways. This event represented a freshwater cyanobacterial bloom spread across an estuary via a man-made canal system. While microscopic, molecular, and toxin analyses identified *Microcystis* as the primary human health threat during this event, cyanobacteria capable of producing saxitoxins were concurrently present. Levels of microcystin and microcystin synthesis genes paralleled total N levels across the system during the summer. Furthermore, nutrient concentrations, nutrient ratios, and nutrient amendment experiments all indicated that N was the element most capable of promoting algal, and specifically cyanobacterial biomass. Collectively, this study provides important new insights into the controls and toxicity of cyanobacterial blooms along freshwater-to-estuarine gradients.

The biomass of phytoplankton communities across Lake Okeechobee and the St. Lucie River and Estuary was dominated (July 2016) or at least co-dominated (September 2016) by cyanobacteria that were commonly N-limited during this study. Consistent with these findings, DIN:DIP values were below Redfield at almost all sites, a symptom of N-limitation [39]. Though our N and P measurements were limited to our summer and fall transects, our data support the findings of previous studies of this freshwater-estuarine ecosystem, which have shown that both Lake Okeechobee [40–42] and the St. Lucie River and Estuary [43] generally exhibit N-limitation. The absence of nitrogen fixation during our surveys and the dominance of non-nitrogen fixing cyanobacteria observed in our transects is a common symptom of N-limitation in freshwater systems [15], and is consistent with earlier reports of infrequent blooms of diazotrophs in Lake Okeechobee [44]. While we cannot discount the importance of P in affecting algal populations as a co-limiting nutrient at other times, the preponderance of evidence accumulated during this and some prior studies [23, 24, 43] indicates that excessive N loading can promote cyanobacterial and algal populations across Lake Okeechobee and the St. Lucie River and Estuary.

N-limitation in the estuary that subsequently promotes cyanobacterial blooms can also be due to excessive phosphorus loading originating from the North Fork of the St. Lucie River [45], which receives fertilizer inputs from agricultural activities and golf courses [46]. Such inputs may have contributed for the lowest DIN:DIP values of the July and September transects being in the mid-estuary sites SLE 6.5 and SLE 10, and subsequently promoted algal responses to NH_4_^+^ during experiments. Furthermore, LaPointe et al. (2017) emphasized that N emanating from on-site sewage treatment and disposal systems along the St. Lucie River was likely the primary source of excessive nitrogen supporting the 2016 bloom event [47]. As such, the remediation of these systems via upgrading to denitrifying systems or connection to sewage treatment plants would be a logical managerial action to mitigate these events in the future.

**Fig 10:**
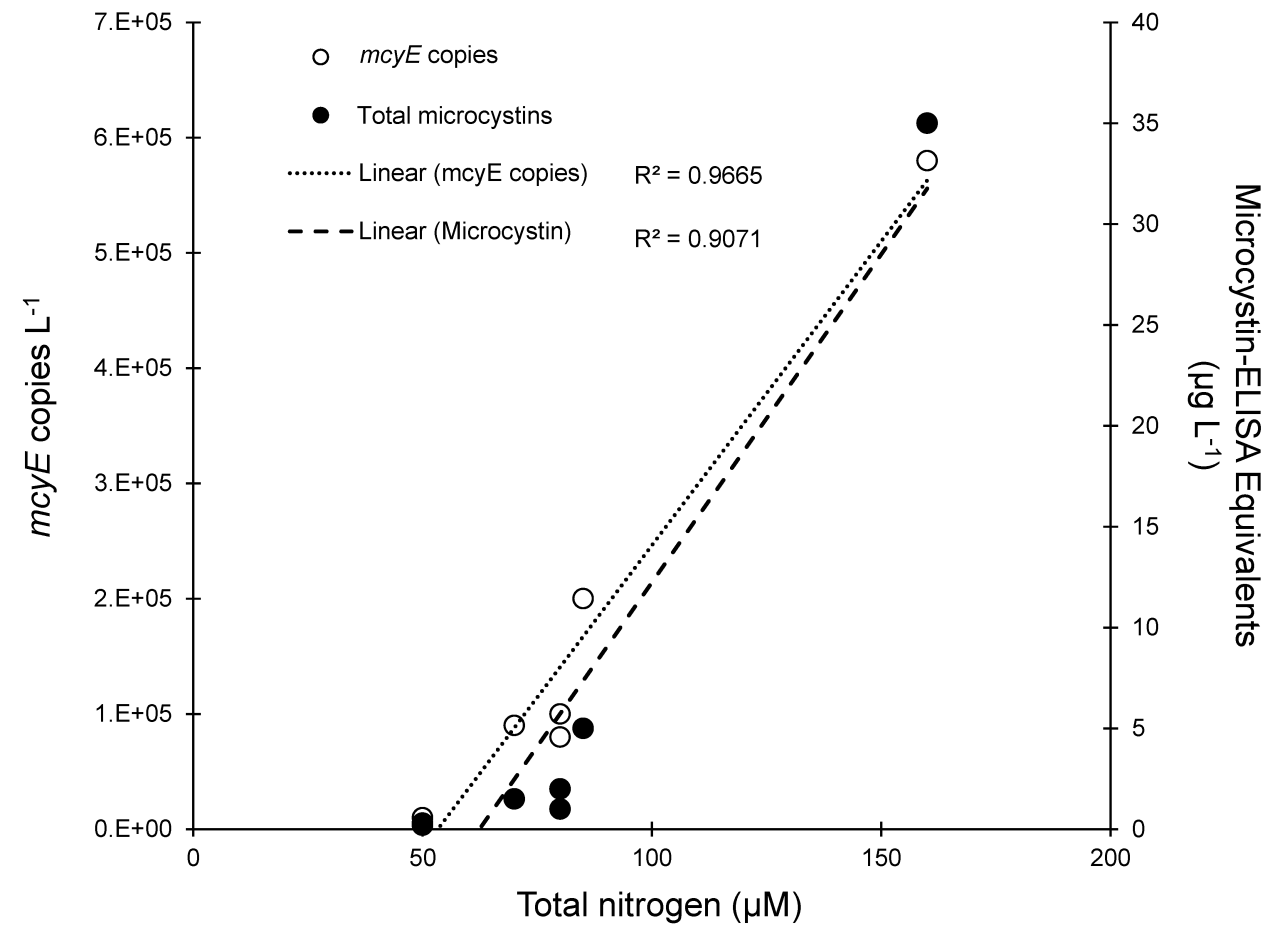
Regression of total nitrogen concentrations, total microcystin concentrations (black circles, dashed regression) and *mcyE* copies (white circles, dotted regression) during the July transect.

Beyond the effects of N on biomass, N seems to have played an important role in controlling the toxicity of the July 2016 event across the Lake Okeechobee—St. Lucie River Estuary gradient, as levels of total nitrogen, microcystin, and *mcyE* gene copies were all highly and significantly correlated with each other (R^2^ > 0.91; *p* < 0.0001 for all; Fig 10). In contrast, microcystin concentrations were not correlated with total *Microcystis* densities, total phosphorus, or inorganic nutrient pools. These findings reinforce several aspects of *Microcystis* eco-toxicology. Firstly, as a N-rich compound, it has been shown in culture studies [48, 49], transcriptomic studies [50, 51], field surveys [10, 15, 52] and field experiments [53] that higher levels of N yield higher levels of microcystin per cell. Furthermore, field and lab studies have shown that higher levels of N favor the dominance of toxic *Microcystis* strains that possess the microcystin synthetase cassette over non-toxic strains lacking these genes [54, 55]. Hence, the tight correlations among microcystin, toxic cells, and total N suggest that this element played a central role in controlling the toxicity of the Lake Okeechobee—St. Lucie River Estuary *Microcystis* bloom. These data also highlight the importance of using molecular methods (e.g. qPCR) to monitor potentially-toxic cell abundances, as these populations have been shown in several studies to better track with toxin concentrations than total cell abundances ([15] and references therein). Given the role that genes such as *ntcA* play in both microcystin synthesis and nitrogen regulation [56–58], other such targets and their co-regulators such as carbon levels and C:N ratios could be quantified to generate a more holistic understanding of N–microcystin interactions.

Oehrle et al. (2017) also collected samples along the St. Lucie River Estuary in early July and reported that NO_3_^-^ levels in the estuary in the months prior to July were elevated relative to non-bloom years [59], suggesting that these high nitrate levels contributed toward the intensification of the bloom during June. The same study used LC-MS/MS to determine that microcystin-LR was the dominant (~ 85%) congener and that levels were within the range reported here for open waters of the SLE (160 - < 1 μg L^-1^) but also found exceedingly higher levels in scums accumulated along shorelines (~ 4000 and ~ 1000 μg L^-1^ in early and mid–July, respectively) in the vicinity of our site SLE 10, where we found 0.5 μg L^-1^ microcystin-ELISA equivalents at this open water site. Shoreline scum samples are known to exhibit significantly higher concentrations of microcystins relative to water samples [60] and the presence of this scum emphasizes the highly serious nature of the human health risk this bloom event posed.

By September, the cyanobacterial bloom along the Lake Okeechobee—St. Lucie River system had diminished with cyanobacteria co-dominating the phytoplankton community at most sites along with green algae and diatoms. Levels of microcystin were lower at this time and correlations between this toxin and other biological and environment variables were not detected. Despite these substantial changes in algal community abundance and composition, N-limitation persisted in the system. Nutrient amendment experiments on samples collected from other freshwater systems indicate that several freshwater phytoplankton groups, including cyanobacteria, green algae, and diatoms, can exhibit N and P co-limitation seasonally [61]. Prior studies have shown freshwater diatoms such as *Stephanodiscus minutulus* and *Asterionella formosa* exhibit N-limitation with respect to biomass and biovolume [61, 62]. Freshwater green algae can also exhibit species-specific responses to nutrient limitation. Yang and Gao (2003), for instance, reported that at elevated CO_2_ levels *Chlorella pyrenoidosa* growth rates are N-limited [63]. Moreover, culture and field-based experiments with *Mougeotia* spp. indicated that this green alga exhibits N and P co-limitation [64]. Ultimately, our results indicate that freshwater cyanobacteria, green algae, and diatoms exhibited N, rather than P, limitation, at several sites along the September transect, an outcome consistent with the low N:P ratios present at that time.

For almost 30 years, cyanobacteria have comprised the majority of phytoplankton biovolume (50–80%) in Lake Okeechobee, likely due in part to excessive P enrichment [11] in the late 20^th^ century that progressively lowered the total N:P from 30:1 to below 15:1 from the mid-1970s to the late 1990s [41]. Historically, cyanobacterial communities in the lake have been dominated by blooms of *Oscillatoria* and *Planktothrix* [65], with infrequent blooms of diazotrophs [44]. The abundance of non-N_2_ fixers relative to N_2_-fixers may be due to the high turbidity and subsequent low irradiance conditions that can be further exacerbated by storm and hurricane activity [66]. Previous work has also shown that *Planktothrix* thrives in low-light environments [67–69]. In addition, *Planktothrix* has also been shown to dominate the phytoplankton community in Sandusky Bay, Lake Erie where turbidity is high and N:P ratios are low [70]. The absence of *Oscillatoria* and minor amounts of *Planktothrix* and the dominance of *Microcystis*, another non-diazotrophic cyanobacteria, in 2016 and during the past decade [24] might be partly explained by the dissolved N:P in the Lake Okeechobee–St. Lucie River and Estuary system, which at nearly all sites was < 15. N-enrichment in this case may have favored the dominance of those cyanobacterial strains that do not fix nitrogen. As Lake Okeechobee is a shallow freshwater system, it is likely that *Microcystis* spp. outcompeted other cyanobacterial taxa due to the availability of regenerated N and P from the sediment, enabling them to dominate the summer algal bloom’s community composition [5, 15].

In addition to nutrients, salinity was likely an important factor in controlling the diversity and toxicity of phytoplankton communities across the Lake Okeechobee and the St. Lucie River Estuary system. During July, site SLE 6.5 and sites further east were brackish (salinity > 3) and coincided with sharp declines in *mcyE* gene copies and *Microcystis* spp. abundance. Chen et al. (2015) reported a negative correlation between salinity and growth rate and pigment concentration in cultures of *Microcystis aeruginosa*. While this is consistent with our findings, the same study found that production of microcystin increased significantly in response to elevated salinity [71], an outcome not observed during this study. However, another strain of *M*. *aeruginosa* was shown to produce lower amounts of microcystin in response to elevated salinity [72], suggesting that production of microcystin in response to changes in salinity is strain-specific.

While cyanobacteria across the Lake Okeechobee—St. Lucie River system possessed a gene important for the synthesis of saxitoxin [73], this toxin was not detected during this study, a finding that could have been related to multiple factors. First, given toxin synthesis is dependent on the full translation of the entire biosynthetic pathway, the presence, but not necessarily transcription and translation, of a single gene in the pathway may not result in the production of the toxin. Furthermore, the ELISA kits used to quantify saxitoxins here have somewhat elevated detection limits and only detect some of the more than 25 congeners of the toxin [74]. A study utilizing high-performance liquid chromatography (HPLC), which is a more robust and sensitive method to detect saxitoxins than the ELISA used in this study (limit of detection = 2 x 10^−5^ μg mL^-1^), found that samples from blooms in several Australian lakes with *sxtA* gene abundances comparable to values reported in this study had very low, but detectable, saxitoxin concentrations ≤ 8.6 x 10^−6^ μg mL^-1^ [75]. This suggests that saxitoxin might have been present in the Lake Okeechobee–St. Lucie River Estuary waterway, albeit below the limit of detection for our method. Furthermore, these results highlight the importance of incorporating molecular techniques into routine monitoring programs, as these methods are sensitive enough to detect the genetic potential to produce toxins, even if toxin concentrations are too low to quantify. This is important as future changes to several established environmental drivers of bloom growth and toxicity including temperature, land use, invasive species, and rainfall patterns [2] may facilitate shifts in bloom dominance. Therefore, monitoring the genetic potential for toxin production is critically needed, especially during blooms where more than one potential toxin producer is present and in systems where the full suite of toxin producers is unknown.

In conclusion, the cyanobacterial bloom found in Lake Okeechobee and throughout the St. Lucie River and Estuary in the summer of 2016 was dominated by *Microcystis* and exhibited N-limitation. In addition, the levels of microcystin and toxic *Microcystis* cells (possessing *mcyE*) were highly correlated to the levels of total N in this system. Though the bloom had diminished by September, multiple phytoplankton groups including cyanobacteria consistently exhibited N-limitation. Collectively, evidence indicates that reductions in N-loading associated with on-site sewage treatment and disposal systems [47] are likely an important managerial step that could minimize the intensity of future toxic *Microcystis*-dominated cyanobacterial blooms across the Lake Okeechobee—St. Lucie River system.

## Supporting information

S1 File Microcystin concentrations (μg L^-1^). (XLSX)

S2 File Chlorophyll *a* concentrations (μg L^-1^). (XLSX)

S3 File Cell density (cells mL^-1^). (XLSX)

S4 File Total nutrient concentration (μM) and ratio. (XLSX)

S5 File Dissolved nutrient concentration (μM) and ratio. (XLSX)

S6 File *Mcy*E and *sxt*A gene abundances (copies mL^-1^) at sites along the July 2016 transect. (XLSX)

S7 File Chlorophyll *a* data (μg L^-1^) from July 2016 24 h nutrient amendment experiments from samples collected from sites LO 1, SLE 1, SLE 6.5, and SLE 10. (XLSX)

S8 File Specific growth rate data (day^-1^) from July 2016 24 h nutrient amendment experiments from samples collected from sites LO 1, SLE 1, SLE 6.5, and SLE 10. (XLSX)

S9 File Microcystin concentrations (μg L^-1^). (XLSX)

S10 File Chlorophyll *a* concentrations (μg L^-1^). (XLSX)

S11 File Cell density (cells mL^-1^). (XLSX)

S12 File Total nutrient concentration (μM) and ratio. (XLSX)

S13 File Dissolved nutrient concentration (μM) and ratio. (XLSX)

S14 File September 2016 diatom, cyanobacteria, and green algal pigment concentrations (μg L^-1^) from all transect sites. (XLSX)

S15 File Chlorophyll *a* data (μg L^-1^) at 24 and 72 h from the September 2016 nutrient amendment experiment. (XLSX)

S16 File Specific growth rate data (day^-1^) at 24 and 72 h from the September 2016 nutrient amendment experiment. (XLSX)

S17 File Diatom, cyanobacteria, and green algal pigment concentrations (μg L^-1^) at 48 h from the September 2016 nutrient amendment experiment. (XLSX)

S18 File Regression of total nitrogen concentrations (μM), total microcystin concentrations (μg L^-1^) and *mcy*E gene abundances (copies mL^-1^) during the July transect. (XLSX)
